# Death of a close friend: Short and long-term impacts on physical, psychological and social well-being

**DOI:** 10.1371/journal.pone.0214838

**Published:** 2019-04-04

**Authors:** Wai-Man Liu, Liz Forbat, Katrina Anderson

**Affiliations:** 1 School of Finance, Actuarial Studies and Statistics, Australian National University, Acton, ACT, Australia; 2 Faculty of Social Sciences, University of Stirling, Scotland, United Kingdom; 3 ANU Medical School, Academic Unit of General Practice, The Canberra Hospital, Garran, ACT, Australia; University of Graz, AUSTRIA

## Abstract

This paper reports the impact of a major life event–death–on the physical, psychological and social well-being of the deceased’s close friends. We utilised data from a large longitudinal survey covering a period of 14 years (2002–2015) consisting a cohort of 26,515 individuals in Australia, of whom 9,586 had experienced the death of at least one close friend. This longitudinal cohort dataset comprises responses to the SF-36 (health related quality of life measure) and allowed for analysis of the short and longer-term impacts of bereavement. In order to manage the heterogeneity of the socio-demographics of respondents who did/not experience a death event, we use a new and robust approach known as the Entropy Balancing method to construct a set of weights applied to the bereaved group and the control group (the group that did not experience death). This approach enables us to match the two groups so that the distribution of socio-demographic variables between the two groups are balanced. These variables included gender, age, marital status, ethnicity, personality traits, religion, relative socio-economic disadvantage, economic resources, and education and occupation and where they resided. The data show, for the first time, a range of negative and enduring consequences experienced by people following the death of a close friend. Significant adverse physical and psychological well-being, poorer mental health and social functioning occur up to four years following bereavement. Bereaved females experienced a sharper fall in vitality, suffered greater deterioration in mental health, impaired emotional and social functioning than the male counterparts up to four years after the death. The data show that the level of social connectedness plays an important role in bereavement outcomes. Specifically, we found that less socially active respondents experienced a longer deterioration in physical and psychological health. Finally, we found evidence that the death of a close friend lowered the respondent’s satisfaction with their health. Since death of friends is a universal phenomenon, we conclude the paper by reflecting on the need to recognise the death of a close friend as a substantial experience, and to offer support and services to address this disenfranchised grief. Recognising bereaved friends as a group experiencing adverse outcomes can be used internationally to prompt health and psychological services to assist this specific group, noting that there may be substantial longevity to the negative sequelae of the death of a friend. Facilitating bereaved people’s support networks may be a fruitful approach to minimising these negative outcomes.

## Introduction

In most circumstances, grief is a natural response to bereavement. As part of the grieving process, people adjust and adapt to their loss, but the level of adaptation, and the corresponding emotional, behavioural, psychological and physical responses differ depending on the bereaved individuals’ age [1–4], ethnicity [5], personality traits [6–8], religiosity [9–12], resilience [13, 14], the support they received [15], and the relationship they have with the deceased [16, 17].

Much of the prior research on bereavement confines the focus to the death of a first-degree relative, often a spouse [14]. Within this body of work, the focus is generally on how bereavement influences the risk of mortality [14, 18], physical health and mental wellbeing of the surviving members [14, 17, 19]. There is an emerging body of work looking at predictors of bereavement outcomes, both theoretically [20] and empirically [21–24]. The latter has been extended to complicated grief which has attracted a lot of attention over the past decade because of its clinical implication for palliative care support [24].

Non-kinship bereavement, however, receives surprisingly little attention in the literature, even though it is a ubiquitous experience. Grieving the death of a friend is an experience which receives less social support because the relationship is of lower status than kin, and hence a form of disenfranchised grief [25, 26]. Non-kin grief may not be openly acknowledged or expressed, and the psychological or physical impacts of the grief may be regarded as illegitimate [26]. Therefore, the impact of bereavement of a friend may be trivialised, or at least not afforded the same status, with diminished right to grieve, as that of kin bereavement.

Existing studies on non-kinship bereavement are limited, and they focus almost exclusively on older adults [27–30]. The finding of these studies are mixed, possibly due to heterogeneity between the bereaved and the non-bereaved group. Their sample size are small, and they lack appropriate control variables to address the sociodemographic differences in the sample. Large scale data sets on non-kin relationships are rare, making such study difficult.

A study by Ackerman and his colleagues explored the question “*Is friendship akin to kinship*?” [31]. Drawing on theories in the psychology and evolutionary biology literature they argued that psychology of friendship may mimic that of kinship [32–34]. Ackerman et al’s findings imply that when a close friend dies, bereavements may be experienced in a way similar to the death of kin. In a large and dense social network, possible reinforcement of distress can reduce the supporting function of the network. For example, when a member of the social network dies, several other members within the network also experience varying levels of distress from bereavement, and this can cause network stress [35]. Presence of network stress implies a positive association between the density of the network and negative bereavement outcomes.

In contrast to the network stress argument, a recent study used data of more than 15,000 Facebook networks and studied the social network response following the death of a close friend [36]. They found interactions among friends of decedents intensified and reached a stable level a year after death. This result suggests that social network exhibits resilience and online social interactions represent a form of social support during and after the acute grieving period.

Building on the findings of the bereavement literature and Ackerman et al, we explored the research question of whether bereavement of a close friend exhibits physical and psychological features similar to the death of kin which has been documented in the bereavement literature. More specifically, we hypothesised that even though a person with a broad social network may experience a greater chance of losing a friend, they tend to cope better with grief because their social network may provide a good source of emotional support through the bereavement process, and companionship protects bereaved individuals from despair and loneliness [15, 37]. This implies that those who are socially isolated or less engaged with social activity are expected to exhibit less resiliency to grief than those who are socially active [38, 39].

## Methods

### Data source

Our data came from The Household, Income and Labour Dynamics in Australia (HILDA) Survey. The survey is funded by the Federal Government and is one of the largest longitudinal surveys in Australia starting in 2001 (wave 1) comprising 7,682 households and 19,914 adults. The last wave (wave 15) comprised 17,606 households and 23,292 adults. Samples size varies across waves due to loss to follow-up death or changes in household composition. Further an additional top-up sample was added in wave 11, of 2,153 households (comprising 5,451 individuals).

### Survey participants

Survey participants are interviewed and followed annually. The survey collects information on household and family relationships, income, employment, health and education. It comprises four sets of questionnaires; (1) a household form, (2) a household questionnaire, (3) a individual questionnaire for all household members aged 15 years and above, and (4) a self-completion questionnaire (SCQ). Questionnaires are collected at a later date or returned by post. The SCQ contains a rich set of questions including 36 items that were subsequently aggregated into 8 scales measuring various domains of well-being called the Short Form 36 (SF-36) (reference for SF-36). Each of these eight scales are transformed by HILDA into an index ranges between 0 and 100, where 0 is poor and 100 is excellent.

### Variables and measures

We utilised the respondents’ SF-36 scores on five domains to measure outcomes of bereavement. The five domains are general health, vitality, mental health, role limitations due to emotional problems and social functioning. A previous study examined the validity of the SF-36 scores reported in the HILDA survey and found that they are comparable to published SF-36 normative data and hence can be interpreted in a similar way [40]. In addition to the SF-36, we included one additional survey item that asked how satisfied respondents were with their health and overall life, ranging from 0 (totally dissatisfied) to 10 (totally satisfied).

Our analysis relied on a specific set of questions in the survey concerning whether and how long ago (0 to 3 months ago, 3–6 months ago, 6–9 months ago, 9–12 months ago) the respondents experienced a “life event”. The life event analysed for this paper was the death of a close friend. The event timing question allowed us to infer the timing of the death event at the quarterly interval. Using this life event as the “treatment variable” we tracked the short-term and long-term impact of this death on the respondents’ physical and psychological well-being, mental health, social functioning and life satisfaction.

Our longitudinal analysis covered survey responses from wave 2 (2002) to wave 15 (2015). Wave 1 was excluded because it did not contain a survey question on death. Household residents who were not the responding person to the interview or did not complete the SCQ for the life event questions were excluded from our sample. Our final sample size per wave varied depending on whether they responded to the life event questions and to other questions surrounding their well-being and activities (see Table 1). Our final sample comprised 26,515 individuals who responded to the life event question on the death of a close friend in one or more waves of data collection. This amounts to a total of 168,104 responses over a 13-year period. Of 26,515 respondents, 9,586 (36%) reported to have experienced the death of a close friend over the past 12 months, which amounts to 18,961 death events.

**Table 1:** Descriptive statistics–full sample.

	Death of close friend in the past year?					
	Yes		No			
	*Mean*	*SD*	*Mean*	*SD*	*Diff*.	*P*-value
*Female*	0.54	0.50	0.53	0.50	0.01	0.03
*Age*	53	19.58	43	17.94	9.88	<0.01
*Aborigines or Torres Strait Islander*	0.0032	0.06	0.0026	0.05	0.00	0.11
*Married*	0.51	0.50	0.49	0.50	0.01	<0.01
*Education*	6.57	2.55	6.04	2.69	0.54	<0.01
*Remote*	0.16	0.37	0.13	0.33	-0.40	0.01
*Personality scale—Agreeableness*	5.51	0.85	5.38	0.83	0.13	<0.01
*Personality scale—Conscientiousness*	5.15	0.95	5.10	0.95	0.06	<0.01
*Personality scale—Emotional stability*	5.32	1.01	5.19	0.98	0.13	<0.01
*Personality scale—Extroversion*	4.49	0.96	4.42	1.01	0.07	<0.01
*Personality scale—Openness to experience*	4.15	1.01	4.21	0.98	-0.07	<0.01
*Religion*	0.75	0.43	0.66	0.47	0.10	<0.01
*Relative socio-economic disadvantage Index* (*decile*)	5.34	2.83	5.71	2.84	-6.09	<0.01
*Index of economic resources* (*decile*)	5.00	2.87	5.52	2.87	0.04	<0.01
*Index of education and occupation* (*decile*)	5.20	2.86	5.65	2.89	-0.36	<0.01
*Level of social activity*	3.37	1.47	3.47	1.48	-0.10	<0.01
*Fraction of respondents with low level of social activity*	0.22	0.41	0.23	0.42	-0.01	<0.01
Observations	18,961		149,143			

This Table presents descriptive statistics of the full sample including, whether the respondents had experienced death of a friend in the past year, and respondents’ demography which includes gender (dummy variable equals 1 for female), age, ATSI (dummy variable equals 1 for Aboriginal or Torres Strait Islander), marital status (dummy variable equals 1 if married), highest education level ranging from 1 (if completed a postgraduate degree) to 9 (if completed year 11 or below), whether the respondent resides in a remote area (dummy equals 1 if residing in outer regional cities, remote and very remote Australia based on the Australian Standard Geographical Classification–Remoteness Area, and 0 if residing in major cities/inner regional cities, and decile index for relative socio-economic disadvantage, economic resources, and education and occupation. Five personality character traits (agreeableness, conscientiousness, emotional stability, extroversion and openness to experience) are summarised. Level of social activity are reported (ranging from 1 (everyday) to 7 (less often than once every 3 months)). Religion is a dummy variable equals 1for any religion.

The data also report a second question about death, specifically the death of a family member (excluding spouse and child) over the past 12 months. Combining the positive responses of this question (19,522) and the positive responses of the death of a close friend (18,961) gives us a combined percentage of 22%, which was almost perfectly in line with national statistics reported in the Australian Bureau of Statistics (ABS) where 22% of Australians experienced the death of a family member or close friend over the past 12 months [41].

A key variable of interest was participants’ level of social engagement. It was based on the survey question probing the extent to which respondents get together socially with friends/relatives that are not living with them (ranging from 1 (everyday) to 7 (less often than once every 3 months)). Respondents were considered to have a low level of social activity if they met socially at most once every month. We redefined low social activity as meeting socially with family/friends at most 2–3 times per month and we obtained qualitatively similar results.

We matched and controlled for the respondents’ socio-demographics including age, gender, personality trait, religion, ethnicity (Aboriginal and Torres Strait Islanders), marital status, urban/rural residence, and their socio-economic status based on the area they lived. HILDA survey respondents were questioned on their personality traits using a 36-item inventory. We also matched and controlled for the respondents’ five personality traits that may have impact on their social engagement and have a moderating effect on depressive symptoms following death, because they relate the psychological responses to loss [42]. These variables also relate individuals’ resiliency, adaptive capacities and the ability to cope in response to stressor [43]. For example, extroverted people may better manage their emotional stress because they are more receptive to support from others. Based on Goldberg’s Big Five personality trait descriptive adjectives approach [44, 45], HILDA reports survey items into five personality character traits: agreeableness, conscientiousness, emotional stability, extroversion and openness to experience. It was reported in waves 5, 9 and 13. Since personality traits are reasonably stable over time, for the purpose of identifying heterogeneity across respondents, we used the average value across the three waves as proxy for each respondent’s personality trait scale. Our results remained largely unchanged when the backfilled values of the respondent’s personality trait scale were used.

Respondents were asked about their religion in waves 4, 7, 10, 14. We created a dummy variable which equalled 1 if the respondent was reported to have any religion and 0 if they have no religion. For non-reporting waves, we backfilled missing values using the first available data. For example, religion data in waves 2 and 3 was backfilled using data from wave 4. For wave 15, data from wave 14 was used.

We also controlled for respondents’ marital status and whether they lived in a remote area. Married couples typically receive support from partners and thus are hypothesised to experience a reduced impact of bereavement.

Finally, based on ABS’s socio-economic indicators for areas (SEIFA) from the 2001 census, we controlled for level of socio-economic disadvantage, the level of economic resources, and education and occupation. The Indices reported in HILDA corresponds to SEIFA scores from the ABS Census data.

### Methodology

One of the challenges in this study was to match the two groups of respondents (those that were bereaved and those that were not) given that there are a multitude of factors affecting the bereavement outcome. Instead of conducting one-to-one matching, we matched both groups according to the data distribution of each respondents’ socio-demographics. In doing so, we employed Entropy Balancing (EB) matching [46]. This procedure is essentially a data pre-processing procedure that *reweights* the control sample to improve the level of *independence* between the treatment variable (bereavement) and the respondents’ socio-demographics. The method involves constructing a set of weights applied to each observation in the control group, i.e. the group that did not experience the death, so that distributional balance (mean, variance and skewness) of covariates between the two groups can be achieved. To obtain the optimal weights *w*_*i*_, the procedure involves minimising Kullback’s entropy divergence [47], *H*(*w*):

$$\underset{w_{i}}{\min}H(w)=\sum_{\left\{i|D=0\right\}}w_{i}\log(w_{i}/q_{i})$$ (1)

subject to:

$$\underset{\left\{i|D=0\right\}}{\sum }w_{i}c_{ri}^{k}(X_{i})=m_{r}^{k},\text{for}\;r\;\in \;1,\ldots ,\;R$$ (2)

$$\underset{\left\{i|D=0\right\}}{\sum w_{i}}=1$$ (3)

wi≥0,for alli (4)

where *q*_*i*_ is the base weight. We defined *q*_*i*_ as the uniform weights, for *q*_*i*_ = 1/*n*_0_. *n*_*0*_ is the number of control group. $\overset{k}{\underset{ri}{c}}(X_{i})$ is the set of balance constraints imposed on the moment *k* of each covariate *X*_*i*_ in the reweighted control group. *D* is dummy variable that equals 0 if the sample belongs to the control group.

In contrast to propensity score matching which is a popular method for estimating the treatment effect in observation studies, EB approach is superior because (i) it does not discard samples with low matching scores so it prevents information loss, and equations (ii) it does not rely on how well the propensity score models are fitted in the first stage to obtain the score weights, as EB directly adjusts the weights to the known sample moments to achieve a high level of covariate balance [46].

EB matching procedure and data analysis were executed using STATA 14 statistical software (StataCorp LP, College Station, TX). Using the weights obtained from EB procedure, we then used the Ordinary Least Squares (OLS) to estimate the treatment effect by regressing the well-being measures against the treatment variable: *DEATH* (a dummy variable that equals 1 if the subject experience the death, and 0 otherwise) and an interaction term to tease out the marginal effect of gender and social activity. For example, if the interaction term: *DEATH*×*LOW SOCIAL ACTIVITY* is negative and statistically significant in the SF-36 outcome regression, bereaved individuals with low social engagement experienced poorer bereavement outcome than bereaved individuals with high social engagement. In our weight-adjusted OLS regressions, we included respondents’ socio-demographics as control variables. Coefficients of the treatment variable, interaction terms, and their corresponding *p*-values (two-tailed *t*-test) were reported. For brevity, we did not report the estimated coefficients of the control variables. Since we are testing five domains of welling being over multiple time points, we applied Benjamini-Hochberg procedure [48] to adjust for a false discovery rate of 5%. The coefficient is considered to be significant if the *p*-value is less than Benjamini-Hochberg critical value. The total number of observations used in this study was 168,104.

## Findings

Table 1 presents the descriptive statistics of respondents’ socio-demographic characteristics separated out by bereaved and non-bereaved groups. It shows that the two groups of respondents were quite different socio-demographically (as *t*-tests were all statistically significant at the 5% level). The two groups were not comparable at baseline so the use of EB procedure to reweight the non-bereaved control group made the groups comparable based on the distribution of their socio-demographic characteristics.

First, there was a slightly greater female concentration in the bereaved group (54% bereaved vs 53% control, *p*-value = 0.03). Compared to the non-bereaved group, the bereaved group was significantly older (53 vs 43, *p*-value < 0.01), less educated, more religious (75% vs 66%, *p*-value < 0.01), resided in a more remote part of Australia and resided in economically disadvantaged areas (i.e. lower income families and people with little training and in unskilled occupations) and lesser educational attainment and skilled occupations (*p*-values < 0.01).

Personality traits of these two groups of respondents also differed statistically. The bereaved group reported higher rates of *agreeableness*, *conscientiousness*, *emotional stability*, *extroversion* but scored lower on the scale of *openness to experience*. At least three of these attributes are associated with broader social network and higher level of social activity, which is in line with lower level of social isolation reported in the last row of Panel A. The bereaved cohort were more emotionally stable, possibly due to the age gap between the two cohorts; older people tend to be more emotionally stable than young people [49].

Tables 2 and 3 present evidence that there was a correlation between gender and social activity. For the bereaved group (Table 2), 80% of female respondents met friends/relatives more than once a month, whereas for males, the corresponding statistic was 4% lower (*p*-value < 0.01). For the non-bereaved group, the corresponding difference was similar. This correlation suggests that females are likely to have a broad social network and thus may receive more bereavement support.

**Table 2:** Level of social activity–bereaved group.

	Female		Male		*Diff*.	*P*-value
							*Mean*	*SD*	*Mean*	*SD*
*Level of social activity*	3.29	1.43	3.47	1.5	-0.18	<0.01
*Fraction of respondents with low level of social activity*	0.20	0.40	0.24	0.43	-0.04	<0.01
Observations	10,219		8,742			

This Table presents descriptive statistics of the level of social activity of the bereaved group sample. Level of social activity are reported (ranging from 1 (everyday) to 7 (less often than once every 3 months)). If the respondents met with their friends/relatives less than or around once a month, their level of social activity is considered to be low.

**Table 3:** Level of social activity–non-bereaved group.

Panel C: Non-Bereaved group	Female		Male		*Diff*.	*P*-value
							*Mean*	*SD*	*Mean*	*SD*
*Level of social activity*	3.40	1.46	3.55	1.49	-0.15	<0.01
*Fraction of respondents with low level of social activity*	0.22	0.41	0.25	0.43	-0.03	<0.01
Observations	79,142		70,001			

This Table presents descriptive statistics of the level of social activity of the non-bereaved group sample. Level of social activity are reported (ranging from 1 (everyday) to 7 (less often than once every 3 months)). If the respondents met with their friends/relatives less than or around once a month, their level of social activity is considered to be low.

**Fig 1:**
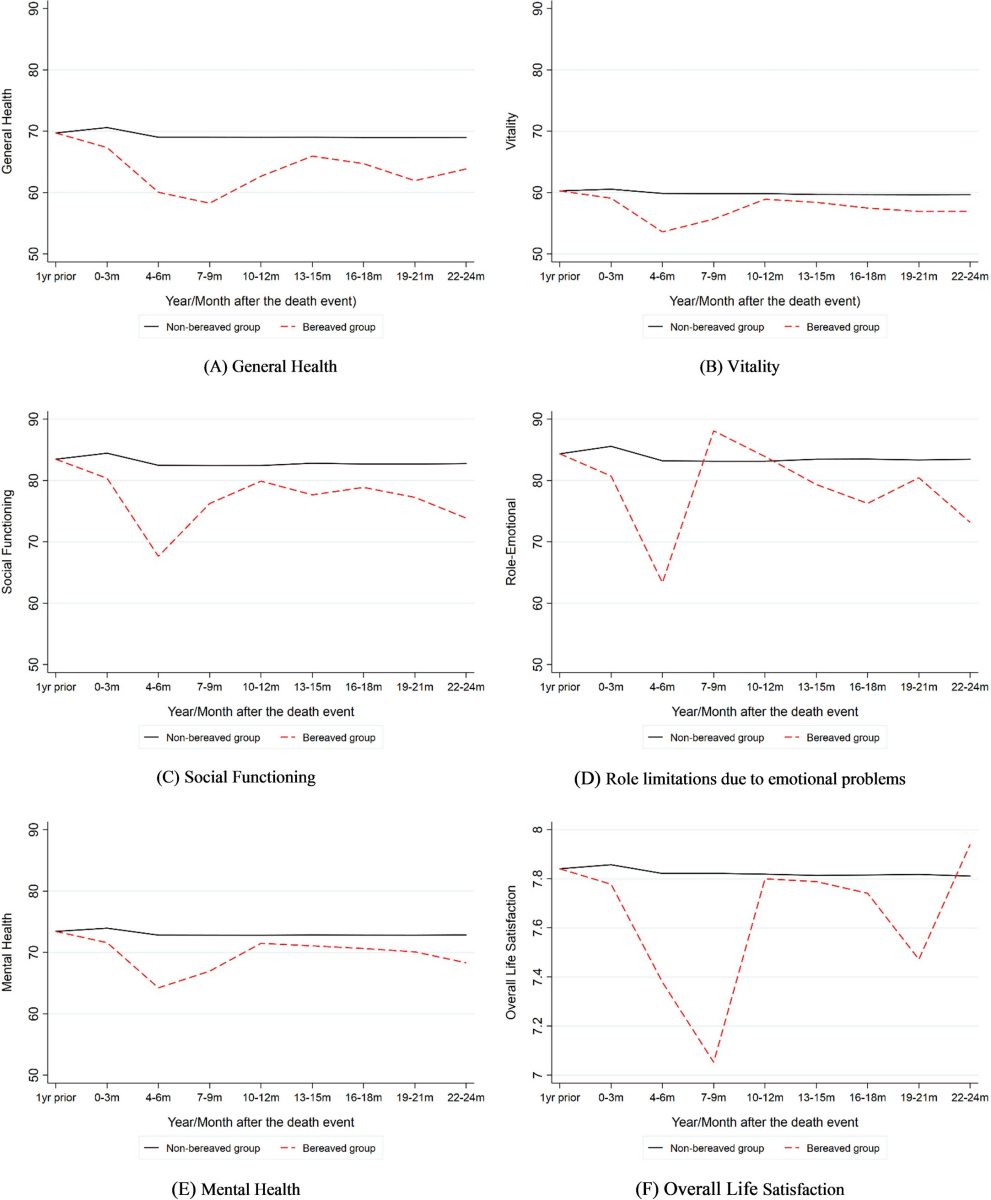
Short term and long term impact of death of a friend over time. This figure presents the impact of the death of a friend on their well-being (separated by gender) over time. The well-being measures include the adjusted mean scores of Short Form 36 Questionnaire (SF-36) scores on the respondent’s general health, vitality, mental health, role limitations due to emotional problems and social functioning. The mean scores were adjusted by respondents’ socio-demographics including age, marital status, ethnicity (ATSI), level of education, remoteness, personality traits, religion, socio-economic disadvantage, economic resources, and education and occupation.

Fig 1 presents the impact of the death on well-being measures from the year prior to death to two years after death across bereaved and non-bereaved respondents. There are two patterns that stand out from the figure. First, after applying the EB procedure to reweight the sample and adjusting for the respondents’ socio-demographics, Fig 1A–1F show that the death of a close friend is *associated* with poorer self-reported general health, lower vitality, poorer mental and psychological well-being, greater interference with normal social activities, greater role limitations caused by emotional problems, and lower life satisfaction following the death. The difference between the two groups at each time point from 0–3 months up until 22–24 months were statistically significant with at the 5% level (*p*-value < 0.01).Note that after reweighting, the first three moments of the distribution (mean, standard deviation and skewness) of all covariates were matched almost perfectly.

Second, there is a notable pattern in well-being metrics and (with the exception of general health) all show marked decline at about 6 months after death, followed by a recovery. Apart from overall life satisfaction, all metrics demonstrate small but varying level of deterioration shortly after recovery.

Table 4 reports the result by the respondent’s social activity. Respondents who were classified as socially inactive (met with friends/family at most once a month) suffered a significant long-term deterioration in vitality, mental health, role-emotional and social functioning, with the greatest impact occurring around 7–9 months after the death. They were significantly less satisfied with their life and health compared with the matched non-bereaved group. For socially active respondents, the greatest impact occurred around the anniversary of death, followed by a steady recovery. While death of a close friend did not seem to decrease their life satisfaction, they were not satisfied with their health up to two years after death, and this corresponds to their overall grief-associated poorer health. Full results of Table 4 can be found in S1 Table.

**Table 4:** Impact of death of a close friend on vitality, mental health, general health, role emotional and social functioning across level of social activity.

	0 to 3 months after		4 to 6 months after		7 to 9 months after		10 to 12 months after		2 year after		3 year after		4 year after	
	Coeff.	*p* value	Coeff.	*p* value	Coeff.	*p* value	Coeff.	*p* value	Coeff.	*p* value	Coeff.	*p* value	Coeff.	*p* value
**Well-Being Measures**														
*General health* (SF-36)														
*DEATH*	-1.36	<0.01*	-2.56	<0.01*	-1.40	<0.01*	-2.53	<0.01*	-1.32	<0.01*	-0.99	<0.01*	-0.95	<0.01*
*DEATH*×*LOW SOCIAL ACTIVITY*	-5.59	<0.01*	-3.06	<0.01*	-6.31	<0.01*	-3.36	<0.01*	-3.72	<0.01*	-3.53	<0.01*	-3.88	<0.01*
*Vitality* (SF-36)														
*DEATH*	-0.31	0.25	-0.59	0.09	-0.41	0.35	-1.59	<0.01*	0.06	0.8	0.52	0.03	0.68	0.01*
*DEATH*×*LOW SOCIAL ACTIVITY*	-4.83	<0.01*	-4.52	<0.01*	-6.25	<0.01*	-4.74	<0.01*	-4.89	<0.01*	-5.2	<0.01*	-5.94	<0.01*
*Mental health* (SF-36)														
*DEATH*	-0.17	0.45	-0.28	0.33	-0.32	0.38	-1.36	<0.01*	-0.09	0.64	0.25	0.22	0.27	0.19
*DEATH*×*LOW SOCIAL ACTIVITY*	-4.68	<0.01*	-5.25	<0.01*	-5.93	<0.01*	-4.43	<0.01*	-5.18	<0.01*	-5.66	<0.01*	-5.34	<0.01*
*Role-emotional* (SF-36)														
*DEATH*	-1.40	<0.01*	-2.44	<0.01*	-0.83	0.27	-3.40	<0.01*	-1.82	<0.01*	-0.99	0.02*	-0.77	0.07
*DEATH*×*LOW SOCIAL ACTIVITY*	-7.09	<0.01*	-7.02	<0.01*	-10.41	<0.01*	-7.48	<0.01*	-5.58	<0.01*	-5.97	<0.01*	-5.25	<0.01*
*Social functioning* (SF-36)														
*DEATH*	-1.36	<0.01*	-1.56	<0.01*	-1.85	<0.01*	-2.58	<0.01*	-1.69	<0.01*	-1.03	<0.01*	-0.65	0.03*
*DEATH*×*LOW SOCIAL ACTIVITY*	-5.90	<0.01	-6.56	<0.01	-8.30	<0.01	-6.65	<0.01	-6.04	<0.01	-6.36	<0.01	-6.02	<0.01
**Satisfaction**														
*How satisfied are you with your life*														
*DEATH*	0.04	0.03	-0.03	0.26	0.00	0.99	-0.07	0.02*	0.02	0.32	0.08	<0.01*	0.08	<0.01*
*DEATH*×*LOW SOCIAL ACTIVITY*	-0.32	<0.01*	-0.38	<0.01*	-0.42	<0.01*	-0.26	<0.01*	-0.24	<0.01*	-0.34	<0.01*	-0.29	<0.01*
*How satisfied are you with your health*														
*DEATH*	-0.05	0.06	-0.20	<0.01*	-0.13	<0.01*	-0.19	<0.01*	-0.09	<0.01*	-0.03	0.15	-0.03	0.21
*DEATH*×*LOW SOCIAL ACTIVITY*	-0.43	<0.01*	-0.43	<0.01*	-0.54	<0.01*	-0.42	<0.01*	-0.29	<0.01*	-0.34	<0.01*	-0.3	<0.01*

This table presents the weighted OLS regression result on the difference between numerous measures capturing the respondents’ vitality level, mental health, and general health, role emotional and social functioning after matching groups of respondents who had experienced death of a friend in the past year against the respondents’ socio-demographics including age, marital status, ethnicity (ATSI), level of education, remoteness, personality traits, religion, socio-economic disadvantage, economic resources, and education and occupation. Non-bereaved group is reweighted using the Entropy Balancing (EB) procedure so that the distribution (mean, variance and skewness) of the socio-demographic variables are matched to the bereaved group. The dependent variables include the Short Form 36 Questionnaire (SF-36) scores on the respondent’s vitality, mental health, general health, role limitations due to emotional problems and social functioning (transformed into a scale from 0 to 100, where 0 is poor and 100 is excellent), how satisfied they are with their life and health (ranging from 0 to 10). In order to isolate the interdependency between gender and social activity, we also match respondent’s gender in addition to the respondents’ socio-demographics. We report the coefficient of the dummy variable *DEATH*, which equals 1 if the respondent experienced death in the relevant time period. We also report coefficient of the interaction variable *DEATH*×*LOW SOCIAL ACTIVITY*, where *LOW SOCIAL ACTIVITY* is a dummy variable equals 1 if the respondent was reported to meet family and friends socially *at most* once every month.

Asterisk (*) is inserted next to the reported *p*-value if it is less than Benjamini-Hochberg critical value.

Ackerman et al report that females respond to friends as though they are their kin more than the male counterparts. Table 5 shows that bereaved females consistently experienced lower vitality, suffered greater deterioration in mental health, suffered greater limitation on their routine activities and social functioning than bereaved males as the coefficients of the interaction term are negative and statistically significant across all time periods. In contrast to the spousal bereavement literature suggesting that mental health tends to improve over time and approaches to the normal level by the 4^th^ year [50], we show that there exists an interesting U-shaped pattern; the impact declines after the first 3 months and reaches the lowest level at around 7–9 months and rises through to the 4^th^ year after death. Our result suggests that females experienced longer term deterioration of physical and psychological well-being following the death of a close friend. Our data may reflect hypotheses that females share tighter and had greater socioemotional bonds than their male counterparts [51–53]. Full results of Table 5 can be found in S2 Table.

**Table 5:** Impact of death of a close friend on vitality, mental health, general health, role emotional and social functioning across gender.

	0 to 3 months after		4 to 6 months after		7 to 9 months after		10 to 12 months after		2 year after		3 year after		4 year after	
	Coeff.	*p* value	Coeff.	*p* value	Coeff.	*p* value	Coeff.	*p* value	Coeff.	*p* value	Coeff.	*p* value	Coeff.	*p* value
**Well-Being Measures**														
*General health* (SF-36)														
*DEATH*	-1.78	<0.01*	-2.56	<0.01*	-2.03	<0.01*	-3.27	<0.01*	-1.09	<0.01*	-0.68	0.03*	-0.81	0.01*
*DEATH*×*FEMALE*	-1.38	0.01*	-1.24	0.07	-1.39	0.10	0.02	0.98	-2.03	<0.01*	-2.10	<0.01*	-1.99	<0.01*
*Vitality* (SF-36)														
*DEATH*	1.62	<0.01*	0.70	0.12	0.53	0.35	-0.60	0.27	2.24	<0.01*	2.33	<0.01*	2.78	<0.01*
*DEATH*×*FEMALE*	-5.41	<0.01*	-4.27	<0.01*	-4.41	<0.01*	-3.76	<0.01*	-6.14	<0.01*	-5.63	<0.01*	-6.61	<0.01*
*Mental health* (SF-36)														
*DEATH*	0.83	0.01*	0.35	0.36	-0.18	0.72	-0.82	0.07	0.94	<0.01*	1.05	<0.01*	1.31	<0.01*
*DEATH*×*FEMALE*	-3.64	<0.01*	-3.31	<0.01*	-2.75	<0.01*	-2.80	<0.01*	-4.14	<0.01*	-3.94	<0.01*	-4.33	<0.01*
*Role-emotional* (SF-36)														
*DEATH*	-0.72	0.26	-2.14	0.01*	-1.62	0.10	-4.00	<0.01*	0.23	0.65	1.13	0.03*	1.43	<0.01*
*DEATH*×*FEMALE*	-3.98	<0.01*	-3.39	<0.01*	-2.78	0.05	-1.90	0.17	-6.19	<0.01*	-6.54	<0.01*	-6.44	<0.01*
*Social functioning* (SF-36)														
*DEATH*	-0.20	0.65	-1.47	0.01*	-1.75	0.01*	-2.65	<0.01*	-0.30	0.41	0.19	0.61	0.87	0.02*
*DEATH*×*FEMALE*	-4.41	<0.01*	-2.82	<0.01*	-3.65	<0.01*	-2.58	0.01*	-5.15	<0.01*	-5.02	<0.01*	-5.54	<0.01*
**Satisfaction**														
*How satisfied are you with your life*														
*DEATH*	0.00	0.90	-0.09	0.01*	-0.13	<0.01*	-0.11	0.01*	-0.01	0.68	0.01	0.55	0.04	0.06
*DEATH*×*FEMALE*	-0.04	0.26	-0.04	0.39	0.07	0.22	-0.05	0.43	-0.08	0.01	-0.06	0.04*	-0.09	<0.01*
*How satisfied are you with your health*														
*DEATH*	-0.03	0.37	-0.26	<0.01*	-0.17	<0.01*	-0.27	<0.01*	-0.04	0.22	-0.01	0.65	0.01	0.68
*DEATH*×*FEMALE*	-0.20	<0.01*	-0.06	0.37	-0.14	0.08	-0.01	0.86	-0.25	<0.01*	-0.22	<0.01*	-0.25	<0.01*

This table presents the weighted OLS regression result on the difference between numerous measures capturing the respondents’ vitality level, mental health, and general health, role emotional and social functioning after matching groups of respondents who had experienced death of a friend in the past year against the respondents’ socio-demographics including age, marital status, ethnicity (ATSI), level of education, remoteness, personality traits, religion, socio-economic disadvantage, economic resources, and education and occupation. Non-bereaved group is reweighted using the Entropy Balancing (EB) procedure so that the distribution (mean, variance and skewness) of the socio-demographic variables are matched to the bereaved group. The dependent variables include the Short Form 36 Questionnaire (SF-36) scores on the respondent’s vitality, mental health, general health, role limitations due to emotional problems and social functioning (transformed into a scale from 0 to 100, where 0 is poor and 100 is excellent), how satisfied they are with their life and health (ranging from 0 to 10). In order to isolate the interdependency between gender and social activity, we also match the level of social activity in addition to the respondents’ socio-demographics. We report the coefficient of the dummy variable *DEATH*, which equals 1 if the respondent experienced death in the relevant time period. We also report coefficient of the interaction variable *DEATH*×*FEMALE*, where *FEMALE* is a dummy variable equals 1 if the respondent is a female.

Asterisk (*) is inserted next to the reported *p*-value if it is less than Benjamini-Hochberg critical value.

Finally, we found that the impact on males was comparatively smaller. The positive and significant coefficients in year 4 (and some in years 3 and 2) suggest that bereaved males experienced long term improvement in vitality, mental health, role-emotional and social functioning. Our life satisfaction regression indicates that bereaved males were more dissatisfied with their life and health in the short term (up to 1 year) while bereaved females were more dissatisfied in the long term (2 year and beyond). It is not clear whether their dissatisfaction was partly due to long term grief-associated outcome.

## Discussion

Our results offer, for the first time, insight into the bio-psychosocial impacts of the death of a close friend. The data are significant in establishing bereaved friends as an important cohort who may require physical and emotional support in the four years following the death. The data are drawn from a nationally representative sample of Australian households, with a sample size of 9,586 people who had experienced the death of at least one close friend making this the largest study of its kind to date.

The data show that the bereaved and the non-bereaved cohort have very different sociodemographic characteristics. Specifically, the bereaved group was older, less educated, more religious, and they lived in areas of socio-economic disadvantage, lower economic resources, lesser education attainment and skilled occupation. Existing works shows that younger bereaved people present with more pronounced grief than older counterparts, but older people tend to experience greater loneliness [1, 3, 4]. The literature shows that religion in general has a positive impact on grief as it helps individuals to handle major crises such as death, and religious communities often provide social support to help individuals cope with their loss [9, 12, 54]. Different personality traits will impact adaptation during bereavement. Individuals with higher self-esteem are more capable of withstanding stress [55]. Positive emotions buffer individuals against anxiety and depression resulting from bereavements [7, 8, 56]. People who score high on ‘neuroticism’ are more emotionally fragile and they are more likely to experience bereavement maladjustment [57].

Personality traits also affect people’s social activity. People who are agreeable tend to behave altruistically and are more pro-social [58] and thus they are more likely to be selected as potential friends [59]. However, it is also possible that bereavement triggers other friends to rally round and be in contact with them, leading to an increase in social network activity [36]. Extroverted people engage in more social activities as they are more likely to experience positive affect from social activity [58], and therefore they are more motivated to make new friends. People who are not conscientious are expected to exhibit poorer self-control and are more likely to engage in prejudiced behaviour and poorer interpersonal interactions [59–61]. However, some studies found little evidence of the association between conscientiousness and the size of friendship network because being conscientious may lead to higher peer acceptance and helps with maintaining friendship rather than making new friends [59].

After matching and controlling for the sociodemographic differences between the bereaved and non-bereaved groups, we found evidence supporting the argument that greater social engagement can harness more support throughout the bereavement process. For those who were socially isolated or have less social engagement, they were less resilient to grief and the impact could last at least four years. This result differs from recent research [36], which suggested that friends of a decedent become more engaged with each other. Further analysis revealed that females experienced greater negative bereavement outcomes, which may be attributed to gender difference in psychological kinship; females share tighter and had greater socioemotional bonds than their male counterparts [51–53]. Results also showed that males had lower life satisfaction in the short-run (within the first year of the death) and while females had less life satisfaction in the long run (2 years and beyond after the death), and this may be associated with prolonged impact of bereavement in females.

Our results present an alternative explanation for the gender difference in bereavement outcomes. Studies show that mortality risk is higher for widowers than widows [14, 62], but widows are more likely to become depressed [63–65]. Research shows that higher baseline levels of depressive symptoms among females may predispose them to higher risk of developing grief-related depressive disorder [64]. However, it is not clear whether widows were employing more effective coping strategies in dealing with grief than widowers [66]. Others argued that males are more vulnerable to depression following widowhood because they suffer more social deficit [67].

Death of a friend is a universal human experience. Yet friends rank low on the grief hierarchy, and friends are considered lesser mourners than relatives.[68] The long-lasting bereavement outcomes of friends found in our data have international significance, in advocating to disrupt bereavement of a friend as a disenfranchised grief. If the death of a friend continues unchallenged in this category, then it will be all the more difficult for the grievers to overcome the emotional and physical impact of bereavement.

We provide evidence from a robust analysis of a large and representative dataset, for alerting bereavement services and the medical system to consider those most likely to experience adverse bio-psycho-social consequences to the death of a friend. Perhaps, in the absence of complex grief [21], a useful focus could be on connecting people to social networks or religious communities to harness emotional support. Such support is essential for fostering better bereavement adjustment, reducing the likelihood of developing prolonged depressive and physical symptoms. This also connects with the move toward community based approaches to death, dying and bereavement [69]. Specifically, an awareness by general practitioners and primary care health practitioners of the potential vulnerability of bereaved people up to four years post loss could allow them to be more proactive in recognition of grief-related presentations and providing subsequent support.

There are several limitations to our study. First, since our results rely on data from self-completion questionnaires, we may overestimate the gender effect due to self-reporting bias; females may be more willing to recognise and acknowledge health problems than males. Second, we were unable to determine from the data whether respondents developed uncomplicated or complicated grief, which has clinical significance, particularly following its inclusion as a new diagnostic criteria in DSM-V [14, 70]. Third, the data do not provide detailed information about the nature and causes of death which can be important risk factors driving the intensity of grief [71, 72]. For example, violent death [73, 74], unnatural death [75] and unexpectedness or unpreparedness of death [37, 76, 77] can increase the chance of developing complicated grief, and hence some of the bio-psychosocial impacts reported in this paper.

## Conclusion

Bereavement often leads to psychological distress, yet to date there has been a lack of robust data which demonstrates the impact following the death of a close friend. Friends’ responses to bereavement are influenced by the person’s age, gender, race, religion, intrapersonal factors such as personality and mental health.

Bereavement of a close friend is a type of disenfranchised grief. It renders significant negative impact on people’s physical health, vitality, mental health, social functioning and role limitations due to emotional problems. Drawing on the theory of psychological kinship we demonstrated that bereaved females experience more negative and long-lasting bereavement outcomes. Specifically, they experience a substantial fall in vitality, suffered more deterioration in mental health, impaired role-emotional and social functioning than male counterparts for up to four years. Our results showed that people who were not socially active suffered significant adverse physical and psychological well-being, inferior mental health and social functioning. The death of a close friend reduced a person’s social network and interaction. For respondents who were socially engaged, they received support from other friends and relatives during bereavement, and thus the negative impact was somewhat moderated. Overall, we presented robust evidence that the death of a close friend matters. The findings have international applicability regarding the impact of bereavement on close friends. The data suggest the need to ensure services are able to assist people who have experienced the death of a friend to develop support networks.

## Supporting information

S1 Table Impact of death of a close friend on vitality, mental health, general health, role emotional and social functioning across level of social activity (full results). This table presents the weighted OLS regression result on the difference between numerous measures capturing the respondents’ vitality level, mental health, and general health, role emotional and social functioning after matching groups of respondents who had experienced death of a friend in the past year against the respondents’ socio-demographics including age, marital status, ethnicity (ATSI), level of education, remoteness, personality traits, religion, socio-economic disadvantage, economic resources, and education and occupation. Non-bereaved group is reweighted using the Entropy Balancing (EB) procedure so that the distribution (mean, variance and skewness) of the socio-demographic variables are matched to the bereaved group. The dependent variables include the Short Form 36 Questionnaire (SF-36) scores on the respondent’s vitality, mental health, general health, role limitations due to emotional problems and social functioning (transformed into a scale from 0 to 100, where 0 is poor and 100 is excellent), how satisfied they are with their life and health (ranging from 0 to 10). In order to isolate the interdependency between gender and social activity, we also match respondent’s gender in addition to the respondents’ socio-demographics. We report the coefficient of the dummy variable *DEATH*, which equals 1 if the respondent experienced death in the relevant time period. We also report coefficient of the interaction variable *DEATH*×*LOW SOCIAL ACTIVITY*, where *LOW SOCIAL ACTIVITY* is a dummy variable equals 1 if the respondent was reported to meet family and friends socially *at most* once every month. *t*-statistics are reported in parenthesis.
(DOCX)

S2 Table Impact of death of a close friend on vitality, mental health, general health, role emotional and social functioning across Gender (full results). This table presents the weighted OLS regression result on the difference between numerous measures capturing the respondents’ vitality level, mental health, and general health, role emotional and social functioning after matching groups of respondents who had experienced death of a friend in the past year against the respondents’ socio-demographics including age, marital status, ethnicity (ATSI), level of education, remoteness, personality traits, religion, socio-economic disadvantage, economic resources, and education and occupation. Non-bereaved group is reweighted using the Entropy Balancing (EB) procedure so that the distribution (mean, variance and skewness) of the socio-demographic variables are matched to the bereaved group. The dependent variables include the Short Form 36 Questionnaire (SF-36) scores on the respondent’s vitality, mental health, general health, role limitations due to emotional problems and social functioning (transformed into a scale from 0 to 100, where 0 is poor and 100 is excellent), how satisfied they are with their life and health (ranging from 0 to 10). In order to isolate the interdependency between gender and social activity, we also match the level of social activity in addition to the respondents’ socio-demographics. We report the coefficient of the dummy variable DEATH, which equals 1 if the respondent experienced death in the relevant time period. We also report coefficient of the interaction variable DEATH×FEMALE, where FEMALE is a dummy variable equals 1 if the respondent is a female. *t*-statistics are reported in parenthesis.
(DOCX)
