# Correction: Death of a close friend: Short and long-term impacts on physical, psychological and social well-being

**DOI:** 10.1371/journal.pone.0218026

**Published:** 2019-05-31

**Authors:** Wai-Man Liu, Liz Forbat, Katrina Anderson

A reference is incorrectly omitted from the penultimate sentence of the Survey participants subsection in the Methods. The correct sentence is: The SCQ contains a rich set of questions including 36 items that were subsequently aggregated into 8 scales measuring various domains of well-being called the Short Form 36 (SF-36) (Ware & Sherbourne, 1992).

The correct reference is: Ware J, Sherbourne C. The MOS 36-ltem Short-Form Health Survey (SF-36). Medical Care. 1992;30(6):473–483.
