# Supplementary material for: Death of a close friend: Short and long-term impacts on physical, psychological and social well-being
Source: PLoS One. 2019 Apr 4;14(4):e0214838. doi: 10.1371/journal.pone.0214838 (PMC6448887; doi:10.1371/journal.pone.0214838)
Supplement: S2 Table — This table presents the weighted OLS regression result on the difference between numerous measures capturing the respondents’ vitality level, mental health, and general health, role emotional and social functioning after matching groups of respondents who had experienced death of a friend in the past year against the respondents’ socio-demographics including age, marital status, ethnicity (ATSI), level of education, remoteness, personality traits, religion, socio-economic disadvantage, economic resources, and education and occupation. Non-bereaved group is reweighted using the Entropy Balancing (EB) procedure so that the distribution (mean, variance and skewness) of the socio-demographic variables are matched to the bereaved group. The dependent variables include the Short Form 36 Questionnaire (SF-36) scores on the respondent’s vitality, mental health, general health, role limitations due to emotional problems and social functioning (transformed into a scale from 0 to 100, where 0 is poor and 100 is excellent), how satisfied they are with their life and health (ranging from 0 to 10). In order to isolate the interdependency between gender and social activity, we also match the level of social activity in addition to the respondents’ socio-demographics. We report the coefficient of the dummy variable DEATH, which equals 1 if the respondent experienced death in the relevant time period. We also report coefficient of the interaction variable DEATH×FEMALE, where FEMALE is a dummy variable equals 1 if the respondent is a female. t-statistics are reported in parenthesis. (DOCX) [file pone.0214838.s002.docx]

**S2 Table**: Impact of Death of a Close Friend on Vitality, Mental Health, General Health, Role Emotional and Social Functioning Across Gender (full results)

Panel A: 0-3 months after

|  | (1) | (2) | (3) | (4) | (5) | (6) | (7) |
| --- | --- | --- | --- | --- | --- | --- | --- |
|  | *General health*  (SF-36) | *Vitality*  (SF-36) | *Mental health*  (SF-36) | *Role-emotional*  (SF-36) | *Social functioning* (SF-36) | *Satisfied with*  *Life?* | *Satisfied with health?* |
|  |  |  |  |  |  |  |  |
| *DEATH* | -1.78 | 1.62 | 0.83 | -0.72 | -0.20 | -0.00 | -0.03 |
|  | (-4.58) | (4.53) | (2.77) | (-1.13) | (-0.45) | (-0.13) | (-0.89) |
| *DEATH*×*FEMALE* | -1.38 | -5.41 | -3.64 | -3.98 | -4.41 | -0.04 | -0.20 |
|  | (-2.59) | (-11.09) | (-8.90) | (-4.45) | (-7.22) | (-1.12) | (-4.10) |
| *Age* | -0.30 | -0.14 | -0.00 | -0.34 | -0.27 | 0.00 | -0.02 |
|  | (-38.87) | (-18.90) | (-0.13) | (-24.62) | (-28.79) | (5.44) | (-26.53) |
| *Married* | 1.94 | 1.68 | 2.39 | 6.68 | 4.92 | 0.26 | 0.15 |
|  | (6.71) | (6.32) | (10.88) | (13.64) | (14.79) | (12.93) | (5.32) |
| *Remote* | 1.01 | 0.91 | 0.86 | 0.71 | 0.83 | 0.17 | 0.13 |
|  | (2.51) | (2.50) | (2.79) | (1.02) | (1.80) | (6.78) | (3.43) |
| *Relative socio-economic disadvantage Index* (*decile*) | 0.84 | 0.57 | 0.60 | 0.82 | 1.02 | 0.06 | 0.07 |
|  | (7.97) | (5.79) | (7.15) | (4.56) | (8.22) | (7.95) | (7.50) |
| *Index of economic resources* (*decile*) | -0.26 | -0.30 | -0.30 | -0.14 | -0.25 | -0.04 | -0.04 |
|  | (-2.92) | (-3.59) | (-4.16) | (-0.96) | (-2.40) | (-6.87) | (-4.61) |
| *Index of education and occupation* (*decile*) | 0.27 | 0.30 | 0.04 | 0.27 | 0.16 | 0.01 | 0.04 |
|  | (2.63) | (3.11) | (0.49) | (1.54) | (1.30) | (1.27) | (3.72) |
| *Personality scale - Agreeableness* | 0.50 | -0.29 | 0.49 | -1.41 | -0.41 | 0.15 | 0.03 |
|  | (2.62) | (-1.63) | (3.24) | (-4.19) | (-1.80) | (10.96) | (1.46) |
| *Personality scale - Conscientiousness* | 2.36 | 2.15 | 1.28 | 4.01 | 2.62 | 0.08 | 0.18 |
|  | (13.95) | (13.93) | (9.87) | (14.34) | (13.30) | (6.89) | (11.35) |
| *Personality scale - Emotional stability* | 4.60 | 4.30 | 5.87 | 6.64 | 4.72 | 0.23 | 0.32 |
|  | (27.63) | (27.80) | (44.01) | (23.41) | (24.17) | (19.85) | (19.32) |
| *Personality scale - Extroversion* | 2.09 | 2.72 | 2.05 | 1.09 | 1.46 | 0.12 | 0.15 |
|  | (13.82) | (19.43) | (16.97) | (4.35) | (8.28) | (11.99) | (10.40) |
| *Personality scale - Openness to experience* | -0.29 | -0.53 | -0.46 | -1.26 | -1.24 | -0.08 | -0.04 |
|  | (-1.80) | (-3.60) | (-3.63) | (-4.70) | (-6.60) | (-7.43) | (-2.84) |
| *Religion* | 0.49 | 0.30 | -0.17 | -1.51 | -1.15 | 0.03 | 0.03 |
|  | (1.51) | (0.99) | (-0.65) | (-2.80) | (-3.04) | (1.47) | (1.10) |
| *Aborigines or Torres Strait Islander* | 6.13 | 7.47 | 3.31 | 1.27 | 1.62 | 0.49 | 0.72 |
|  | (2.90) | (4.81) | (1.84) | (0.24) | (0.71) | (3.15) | (4.48) |
| *Education* | -0.37 | -0.12 | -0.25 | -0.67 | -0.38 | 0.03 | 0.00 |
|  | (-6.68) | (-2.30) | (-5.64) | (-7.11) | (-5.84) | (8.65) | (0.21) |
| Constant | 31.43 | 21.26 | 25.76 | 48.09 | 52.98 | 4.64 | 4.46 |
|  | (21.86) | (15.96) | (22.59) | (19.77) | (31.39) | (45.44) | (32.26) |
|  |  |  |  |  |  |  |  |
| Observations | 140,599 | 141,689 | 141,682 | 140,451 | 142,203 | 142,220 | 142,228 |
| Adj. R-squared | 0.1363 | 0.1119 | 0.1889 | 0.0874 | 0.1066 | 0.0979 | 0.0775 |

Panel B: 4-6 months after

|  | (1) | (2) | (3) | (4) | (5) | (6) | (7) |
| --- | --- | --- | --- | --- | --- | --- | --- |
|  | *General health*  (SF-36) | *Vitality*  (SF-36) | *Mental health*  (SF-36) | *Role-emotional*  (SF-36) | *Social functioning* (SF-36) | *Satisfied with*  *Life?* | *Satisfied with health?* |
|  |  |  |  |  |  |  |  |
| *DEATH* | -2.56 | 0.70 | 0.35 | -2.14 | -1.47 | -0.09 | -0.26 |
|  | (-5.34) | (1.54) | (0.91) | (-2.63) | (-2.61) | (-2.56) | (-5.48) |
| *DEATH*×*FEMALE* | -1.24 | -4.27 | -3.31 | -3.39 | -2.82 | -0.04 | -0.06 |
|  | (-1.83) | (-6.74) | (-6.21) | (-2.91) | (-3.58) | (-0.86) | (-0.90) |
| *Age* | -0.31 | -0.14 | -0.02 | -0.39 | -0.27 | 0.00 | -0.02 |
|  | (-32.21) | (-14.71) | (-2.63) | (-22.10) | (-23.79) | (3.55) | (-20.17) |
| *Married* | 2.41 | 1.82 | 3.09 | 8.20 | 5.43 | 0.31 | 0.18 |
|  | (6.62) | (5.37) | (11.09) | (13.12) | (12.74) | (11.94) | (5.09) |
| *Remote* | 1.81 | 0.84 | 1.57 | 2.06 | 1.96 | 0.17 | 0.19 |
|  | (3.81) | (1.88) | (4.11) | (2.53) | (3.49) | (5.11) | (4.16) |
| *Relative socio-economic disadvantage Index* (*decile*) | 0.46 | 0.41 | 0.46 | 0.67 | 0.72 | 0.05 | 0.05 |
|  | (3.40) | (3.29) | (4.44) | (3.03) | (4.60) | (5.42) | (4.14) |
| *Index of economic resources* (*decile*) | -0.15 | -0.26 | -0.13 | -0.16 | -0.20 | -0.03 | -0.02 |
|  | (-1.26) | (-2.28) | (-1.36) | (-0.83) | (-1.47) | (-3.91) | (-1.91) |
| *Index of education and occupation* (*decile*) | 0.63 | 0.44 | 0.18 | 0.62 | 0.60 | 0.02 | 0.05 |
|  | (4.86) | (3.71) | (1.79) | (2.83) | (4.01) | (1.79) | (4.12) |
| *Personality scale - Agreeableness* | 0.27 | -0.21 | 0.32 | -1.42 | -0.56 | 0.15 | 0.03 |
|  | (1.13) | (-0.97) | (1.68) | (-3.48) | (-1.93) | (8.29) | (1.19) |
| *Personality scale - Conscientiousness* | 2.66 | 2.63 | 1.43 | 4.40 | 2.66 | 0.08 | 0.22 |
|  | (12.73) | (13.55) | (8.77) | (12.32) | (11.19) | (5.02) | (10.74) |
| *Personality scale - Emotional stability* | 4.28 | 3.59 | 5.73 | 6.01 | 4.21 | 0.23 | 0.31 |
|  | (19.90) | (18.28) | (34.14) | (17.03) | (17.11) | (15.88) | (14.62) |
| *Personality scale - Extroversion* | 1.95 | 2.66 | 2.01 | 1.01 | 1.88 | 0.15 | 0.14 |
|  | (10.41) | (14.98) | (13.35) | (3.17) | (8.39) | (11.39) | (7.44) |
| *Personality scale - Openness to experience* | -0.57 | -0.69 | -0.42 | -2.00 | -1.73 | -0.09 | -0.06 |
|  | (-2.65) | (-3.52) | (-2.55) | (-5.72) | (-6.94) | (-6.39) | (-2.91) |
| *Religion* | 0.03 | -0.25 | -1.19 | -2.17 | -1.90 | -0.08 | 0.05 |
|  | (0.08) | (-0.67) | (-3.66) | (-3.23) | (-4.15) | (-2.95) | (1.29) |
| *Aborigines or Torres Strait Islander* | 0.29 | 2.59 | 1.41 | -7.56 | -4.62 | 0.73 | 0.48 |
|  | (0.06) | (1.07) | (0.37) | (-1.27) | (-0.96) | (3.54) | (0.66) |
| *Education* | -0.53 | -0.23 | -0.27 | -0.70 | -0.49 | 0.02 | -0.02 |
|  | (-7.55) | (-3.52) | (-4.95) | (-5.96) | (-5.86) | (5.11) | (-2.83) |
| Constant | 35.74 | 24.09 | 27.28 | 54.39 | 56.51 | 4.62 | 4.48 |
|  | (19.45) | (14.18) | (19.44) | (17.86) | (25.89) | (36.18) | (24.86) |
|  |  |  |  |  |  |  |  |
| Observations | 138,252 | 139,310 | 139,303 | 138,117 | 139,813 | 139,826 | 139,834 |
| Adj. R-squared | 0.1488 | 0.1085 | 0.1908 | 0.0993 | 0.1162 | 0.1042 | 0.0888 |

Panel B: 7-9 months after

|  | (1) | (2) | (3) | (4) | (5) | (6) | (7) |
| --- | --- | --- | --- | --- | --- | --- | --- |
|  | *General health*  (SF-36) | *Vitality*  (SF-36) | *Mental health*  (SF-36) | *Role-emotional*  (SF-36) | *Social functioning* (SF-36) | *Satisfied with*  *Life?* | *Satisfied with health?* |
|  |  |  |  |  |  |  |  |
| *DEATH* | -2.03 | 0.53 | -0.18 | -1.62 | -1.75 | -0.13 | -0.17 |
|  | (-3.51) | (0.93) | (-0.36) | (-1.64) | (-2.47) | (-2.84) | (-2.93) |
| *DEATH*×*FEMALE* | -1.39 | -4.41 | -2.75 | -2.78 | -3.65 | 0.07 | -0.14 |
|  | (-1.66) | (-5.60) | (-4.07) | (-1.96) | (-3.62) | (1.22) | (-1.76) |
| *Age* | -0.31 | -0.14 | -0.03 | -0.38 | -0.28 | 0.00 | -0.02 |
|  | (-25.22) | (-12.00) | (-3.01) | (-18.04) | (-19.40) | (1.82) | (-18.08) |
| *Married* | 2.62 | 2.01 | 2.84 | 7.44 | 5.81 | 0.29 | 0.22 |
|  | (5.91) | (4.78) | (8.02) | (9.83) | (10.95) | (9.33) | (5.10) |
| *Remote* | 0.05 | 0.39 | 0.40 | -1.06 | -0.29 | 0.22 | 0.08 |
|  | (0.07) | (0.66) | (0.79) | (-1.01) | (-0.40) | (5.17) | (1.34) |
| *Relative socio-economic disadvantage Index* (*decile*) | 0.60 | 0.45 | 0.47 | 0.77 | 0.71 | 0.05 | 0.04 |
|  | (3.69) | (2.93) | (3.54) | (2.81) | (3.54) | (4.75) | (2.78) |
| *Index of economic resources* (*decile*) | -0.27 | -0.22 | -0.19 | -0.19 | -0.12 | -0.03 | -0.02 |
|  | (-1.77) | (-1.53) | (-1.54) | (-0.79) | (-0.70) | (-2.72) | (-1.39) |
| *Index of education and occupation* (*decile*) | 0.54 | 0.34 | 0.06 | 0.17 | 0.31 | -0.00 | 0.06 |
|  | (3.39) | (2.35) | (0.47) | (0.64) | (1.58) | (-0.00) | (3.81) |
| *Personality scale - Agreeableness* | 0.69 | -0.05 | 0.68 | 0.61 | -0.05 | 0.17 | 0.06 |
|  | (2.23) | (-0.19) | (2.70) | (1.15) | (-0.14) | (7.60) | (2.07) |
| *Personality scale - Conscientiousness* | 2.36 | 1.90 | 1.20 | 4.02 | 2.42 | 0.10 | 0.17 |
|  | (8.84) | (7.73) | (5.55) | (8.96) | (7.55) | (5.35) | (6.74) |
| *Personality scale - Emotional stability* | 4.47 | 4.56 | 6.04 | 6.41 | 4.99 | 0.25 | 0.36 |
|  | (17.08) | (18.14) | (28.24) | (14.81) | (15.70) | (12.70) | (13.87) |
| *Personality scale - Extroversion* | 1.62 | 2.25 | 1.70 | 0.53 | 1.23 | 0.12 | 0.11 |
|  | (6.79) | (9.45) | (8.46) | (1.30) | (4.31) | (7.21) | (5.01) |
| *Personality scale - Openness to experience* | -0.26 | -0.51 | -0.71 | -2.60 | -1.56 | -0.10 | -0.09 |
|  | (-1.02) | (-2.14) | (-3.39) | (-6.02) | (-5.12) | (-5.58) | (-3.83) |
| *Religion* | -0.72 | -0.41 | -1.23 | -1.86 | -2.29 | -0.04 | -0.02 |
|  | (-1.48) | (-0.89) | (-3.03) | (-2.23) | (-3.95) | (-1.02) | (-0.51) |
| *Aborigines or Torres Strait Islander* | -13.64 | -5.54 | -4.85 | -25.77 | -15.81 | 0.40 | 0.02 |
|  | (-2.03) | (-1.01) | (-0.77) | (-2.17) | (-2.26) | (0.79) | (0.03) |
| *Education* | -0.28 | -0.07 | -0.21 | -0.72 | -0.41 | 0.03 | 0.00 |
|  | (-3.29) | (-0.82) | (-3.07) | (-5.19) | (-4.00) | (5.90) | (0.39) |
| Constant | 33.39 | 22.33 | 29.03 | 50.50 | 55.00 | 4.50 | 4.50 |
|  | (14.34) | (10.43) | (16.04) | (13.10) | (19.52) | (26.59) | (20.71) |
|  |  |  |  |  |  |  |  |
| Observations | 136,884 | 137,917 | 137,911 | 136,742 | 138,415 | 138,428 | 138,435 |
| Adj. R-squared | 0.1402 | 0.1087 | 0.1803 | 0.0953 | 0.1151 | 0.1103 | 0.0898 |

Panel B: 10-12 months after

|  | (1) | (2) | (3) | (4) | (5) | (6) | (7) |
| --- | --- | --- | --- | --- | --- | --- | --- |
|  | *General health*  (SF-36) | *Vitality*  (SF-36) | *Mental health*  (SF-36) | *Role-emotional*  (SF-36) | *Social functioning* (SF-36) | *Satisfied with*  *Life?* | *Satisfied with health?* |
|  |  |  |  |  |  |  |  |
| *DEATH* | -3.27 | -0.60 | -0.82 | -4.00 | -2.65 | -0.11 | -0.27 |
|  | (-5.62) | (-1.10) | (-1.79) | (-3.96) | (-3.86) | (-2.47) | (-4.69) |
| *DEATH*×*FEMALE* | 0.02 | -3.76 | -2.80 | -1.90 | -2.58 | -0.05 | -0.01 |
|  | (0.02) | (-5.03) | (-4.43) | (-1.38) | (-2.73) | (-0.79) | (-0.17) |
| *Age* | -0.32 | -0.14 | -0.01 | -0.36 | -0.25 | 0.00 | -0.02 |
|  | (-28.30) | (-12.24) | (-0.88) | (-18.05) | (-18.47) | (2.05) | (-18.23) |
| *Married* | 2.82 | 1.94 | 3.13 | 8.21 | 6.13 | 0.31 | 0.23 |
|  | (6.64) | (4.75) | (9.26) | (11.02) | (12.16) | (10.24) | (5.47) |
| *Remote* | 1.37 | 0.47 | 1.49 | 1.21 | 0.62 | 0.11 | 0.08 |
|  | (2.38) | (0.87) | (3.50) | (1.22) | (0.92) | (2.49) | (1.35) |
| *Relative socio-economic disadvantage Index* (*decile*) | 0.58 | 0.43 | 0.52 | 1.45 | 1.06 | 0.04 | 0.05 |
|  | (3.71) | (3.00) | (4.27) | (5.34) | (5.84) | (3.53) | (2.94) |
| *Index of economic resources* (*decile*) | -0.33 | -0.32 | -0.14 | -0.33 | -0.31 | -0.03 | -0.02 |
|  | (-2.41) | (-2.51) | (-1.35) | (-1.46) | (-1.96) | (-2.98) | (-1.47) |
| *Index of education and occupation* (*decile*) | 0.72 | 0.44 | 0.04 | 0.06 | 0.23 | 0.01 | 0.06 |
|  | (4.66) | (3.04) | (0.32) | (0.22) | (1.27) | (1.01) | (3.58) |
| *Personality scale - Agreeableness* | 0.34 | -0.48 | 0.51 | -0.79 | -0.62 | 0.17 | 0.03 |
|  | (1.22) | (-1.84) | (2.19) | (-1.59) | (-1.86) | (7.70) | (1.12) |
| *Personality scale - Conscientiousness* | 2.56 | 2.18 | 1.05 | 3.31 | 2.11 | 0.06 | 0.16 |
|  | (10.81) | (9.89) | (5.63) | (8.23) | (7.59) | (3.50) | (6.64) |
| *Personality scale - Emotional stability* | 4.63 | 4.46 | 6.08 | 6.53 | 4.90 | 0.22 | 0.33 |
|  | (19.82) | (19.79) | (32.01) | (15.97) | (17.30) | (12.70) | (13.90) |
| *Personality scale - Extroversion* | 1.89 | 2.46 | 2.16 | 1.44 | 1.80 | 0.12 | 0.15 |
|  | (9.02) | (12.28) | (12.74) | (3.93) | (7.00) | (7.63) | (7.09) |
| *Personality scale - Openness to experience* | -0.26 | -0.45 | -0.39 | -2.08 | -1.12 | -0.07 | -0.05 |
|  | (-1.11) | (-2.00) | (-2.05) | (-5.12) | (-3.96) | (-4.02) | (-2.20) |
| *Religion* | -0.84 | -0.89 | -1.25 | -2.49 | -2.73 | -0.04 | -0.04 |
|  | (-1.89) | (-2.09) | (-3.35) | (-3.16) | (-5.11) | (-1.27) | (-0.83) |
| *Aborigines or Torres Strait Islander* | 8.10 | 7.31 | 2.23 | 9.32 | -1.13 | 0.48 | 0.94 |
|  | (2.25) | (1.24) | (0.38) | (2.91) | (-0.37) | (0.86) | (4.49) |
| *Education* | -0.46 | -0.21 | -0.29 | -0.78 | -0.61 | 0.03 | -0.00 |
|  | (-5.79) | (-2.76) | (-4.50) | (-5.65) | (-6.32) | (5.56) | (-0.42) |
| Constant | 33.18 | 23.54 | 25.57 | 51.43 | 54.90 | 4.78 | 4.58 |
|  | (16.00) | (12.06) | (14.94) | (14.31) | (21.91) | (30.98) | (22.73) |
|  |  |  |  |  |  |  |  |
| Observations | 137,215 | 138,245 | 138,239 | 137,065 | 138,744 | 138,758 | 138,765 |
| Adj. R-squared | 0.1601 | 0.1175 | 0.2026 | 0.1042 | 0.1237 | 0.0865 | 0.0876 |

Panel E: 2 year after

|  | (1) | (2) | (3) | (4) | (5) | (6) | (7) |
| --- | --- | --- | --- | --- | --- | --- | --- |
|  | *General health*  (SF-36) | *Vitality*  (SF-36) | *Mental health*  (SF-36) | *Role-emotional*  (SF-36) | *Social functioning* (SF-36) | *Satisfied with*  *Life?* | *Satisfied with health?* |
|  |  |  |  |  |  |  |  |
| *DEATH* | -1.09 | 2.24 | 0.94 | 0.23 | -0.30 | -0.01 | -0.04 |
|  | (-3.44) | (7.41) | (3.65) | (0.46) | (-0.82) | (-0.41) | (-1.23) |
| *DEATH*×*FEMALE* | -2.03 | -6.14 | -4.14 | -6.19 | -5.15 | -0.08 | -0.25 |
|  | (-4.60) | (-14.68) | (-11.58) | (-8.56) | (-9.95) | (-2.47) | (-5.98) |
| *Age* | -0.37 | -0.13 | -0.08 | -0.29 | -0.29 | -0.01 | -0.04 |
|  | (-38.14) | (-14.59) | (-10.04) | (-17.82) | (-25.88) | (-20.33) | (-39.91) |
| *Married* | 3.25 | 1.34 | 3.62 | 7.45 | 5.96 | 0.36 | 0.26 |
|  | (12.66) | (5.49) | (17.61) | (17.71) | (19.96) | (20.32) | (10.83) |
| *Remote* | 0.57 | 0.58 | 1.19 | 0.32 | 1.26 | 0.14 | 0.05 |
|  | (1.69) | (1.80) | (4.31) | (0.57) | (3.22) | (5.89) | (1.58) |
| *Relative socio-economic disadvantage Index* (*decile*) | 0.33 | 0.18 | 0.41 | 0.38 | 0.50 | 0.05 | 0.05 |
|  | (3.69) | (2.17) | (5.71) | (2.58) | (4.76) | (7.50) | (5.79) |
| *Index of economic resources* (*decile*) | 0.01 | -0.06 | -0.07 | 0.16 | 0.20 | -0.03 | -0.01 |
|  | (0.17) | (-0.86) | (-1.18) | (1.30) | (2.29) | (-5.72) | (-2.02) |
| *Index of education and occupation* (*decile*) | 0.58 | 0.41 | 0.08 | 0.21 | 0.17 | 0.01 | 0.04 |
|  | (6.77) | (5.16) | (1.16) | (1.48) | (1.69) | (1.95) | (5.04) |
| *Personality scale - Agreeableness* | 0.50 | -0.11 | 0.69 | -0.88 | -0.22 | 0.17 | 0.07 |
|  | (3.13) | (-0.74) | (5.05) | (-3.32) | (-1.15) | (14.45) | (4.46) |
| *Personality scale - Conscientiousness* | 2.58 | 2.06 | 1.46 | 3.66 | 2.35 | 0.09 | 0.18 |
|  | (18.15) | (15.36) | (12.63) | (15.73) | (14.18) | (9.34) | (13.43) |
| *Personality scale - Emotional stability* | 4.29 | 4.24 | 5.68 | 5.49 | 4.47 | 0.21 | 0.30 |
|  | (30.87) | (31.61) | (48.85) | (23.61) | (26.63) | (21.36) | (23.00) |
| *Personality scale - Extroversion* | 1.64 | 2.55 | 2.35 | 1.51 | 1.62 | 0.13 | 0.11 |
|  | (13.43) | (21.85) | (23.11) | (7.37) | (11.09) | (15.42) | (9.23) |
| *Personality scale - Openness to experience* | -0.55 | -1.01 | -0.93 | -3.49 | -2.18 | -0.10 | -0.09 |
|  | (-3.95) | (-7.77) | (-8.35) | (-15.50) | (-13.41) | (-10.43) | (-6.65) |
| *Religion* | -0.07 | -0.33 | -1.02 | -1.69 | -2.31 | -0.01 | 0.03 |
|  | (-0.27) | (-1.40) | (-4.98) | (-4.16) | (-7.99) | (-0.83) | (1.38) |
| *Aborigines or Torres Strait Islander* | 0.14 | 2.24 | 2.24 | -1.30 | -2.58 | 0.47 | 0.46 |
|  | (0.05) | (1.00) | (0.83) | (-0.25) | (-0.77) | (2.25) | (1.82) |
| *Education* | -0.53 | -0.08 | -0.17 | -0.44 | -0.48 | 0.01 | -0.03 |
|  | (-11.05) | (-1.88) | (-4.65) | (-5.77) | (-8.86) | (3.96) | (-7.19) |
| Constant | 38.45 | 24.02 | 27.68 | 58.81 | 60.57 | 5.30 | 5.47 |
|  | (30.28) | (20.25) | (27.53) | (28.71) | (41.06) | (59.32) | (46.36) |
|  |  |  |  |  |  |  |  |
| Observations | 101,220 | 101,676 | 101,675 | 101,127 | 102,002 | 108,367 | 108,371 |
| Adj. R-squared | 0.1427 | 0.1150 | 0.1916 | 0.0929 | 0.1212 | 0.0891 | 0.1036 |

Panel F: 3 year after

|  | (1) | (2) | (3) | (4) | (5) | (6) | (7) |
| --- | --- | --- | --- | --- | --- | --- | --- |
|  | *General health*  (SF-36) | *Vitality*  (SF-36) | *Mental health*  (SF-36) | *Role-emotional*  (SF-36) | *Social functioning* (SF-36) | *Satisfied with*  *Life?* | *Satisfied with health?* |
|  |  |  |  |  |  |  |  |
| *DEATH* | -0.68 | 2.33 | 1.05 | 1.13 | 0.19 | 0.01 | -0.01 |
|  | (-2.12) | (7.61) | (4.01) | (2.24) | (0.50) | (0.59) | (-0.45) |
| *DEATH*×*FEMALE* | -2.10 | -5.63 | -3.94 | -6.54 | -5.02 | -0.06 | -0.22 |
|  | (-4.67) | (-13.24) | (-10.89) | (-9.05) | (-9.61) | (-2.03) | (-5.30) |
| *Age* | -0.37 | -0.13 | -0.09 | -0.31 | -0.29 | -0.01 | -0.04 |
|  | (-36.77) | (-13.87) | (-10.86) | (-18.45) | (-24.98) | (-20.83) | (-39.31) |
| *Married* | 3.04 | 1.43 | 3.71 | 7.93 | 6.10 | 0.36 | 0.25 |
|  | (11.60) | (5.81) | (17.85) | (18.82) | (20.26) | (19.84) | (10.38) |
| *Remote* | 0.49 | 0.64 | 1.00 | -0.12 | 1.29 | 0.14 | 0.09 |
|  | (1.39) | (1.95) | (3.51) | (-0.21) | (3.22) | (5.64) | (2.77) |
| *Relative socio-economic disadvantage Index* (*decile*) | 0.50 | 0.24 | 0.44 | 0.56 | 0.59 | 0.05 | 0.05 |
|  | (5.37) | (2.77) | (5.88) | (3.75) | (5.43) | (7.38) | (6.10) |
| *Index of economic resources* (*decile*) | -0.08 | -0.08 | -0.11 | 0.06 | 0.24 | -0.04 | -0.02 |
|  | (-1.03) | (-1.14) | (-1.73) | (0.51) | (2.74) | (-7.62) | (-2.61) |
| *Index of education and occupation* (*decile*) | 0.51 | 0.36 | 0.03 | 0.09 | 0.05 | 0.01 | 0.05 |
|  | (5.84) | (4.45) | (0.44) | (0.67) | (0.52) | (2.35) | (5.57) |
| *Personality scale - Agreeableness* | 0.56 | -0.13 | 0.66 | -1.00 | -0.26 | 0.15 | 0.08 |
|  | (3.45) | (-0.82) | (4.79) | (-3.83) | (-1.33) | (12.68) | (5.14) |
| *Personality scale - Conscientiousness* | 2.40 | 2.08 | 1.45 | 3.62 | 2.32 | 0.10 | 0.17 |
|  | (16.70) | (15.41) | (12.51) | (15.76) | (13.96) | (9.55) | (13.27) |
| *Personality scale - Emotional stability* | 4.17 | 4.19 | 5.71 | 5.31 | 4.42 | 0.22 | 0.30 |
|  | (29.44) | (30.61) | (48.57) | (23.02) | (26.36) | (21.60) | (22.34) |
| *Personality scale - Extroversion* | 1.78 | 2.63 | 2.32 | 1.52 | 1.71 | 0.14 | 0.12 |
|  | (14.15) | (22.06) | (22.78) | (7.58) | (11.61) | (16.41) | (10.38) |
| *Personality scale - Openness to experience* | -0.71 | -1.04 | -0.89 | -3.31 | -2.03 | -0.10 | -0.10 |
|  | (-4.99) | (-7.78) | (-7.75) | (-14.63) | (-12.32) | (-9.81) | (-7.48) |
| *Religion* | -0.07 | -0.26 | -1.01 | -1.84 | -2.07 | 0.00 | 0.05 |
|  | (-0.29) | (-1.09) | (-4.94) | (-4.51) | (-7.13) | (0.09) | (2.27) |
| *Aborigines or Torres Strait Islander* | -2.74 | 1.07 | -4.29 | -7.51 | -6.45 | 0.20 | 0.24 |
|  | (-0.61) | (0.27) | (-1.08) | (-1.22) | (-1.25) | (0.69) | (0.68) |
| *Education* | -0.49 | -0.08 | -0.18 | -0.42 | -0.44 | 0.01 | -0.03 |
|  | (-10.15) | (-1.65) | (-4.61) | (-5.46) | (-7.88) | (2.76) | (-7.78) |
| Constant | 39.54 | 23.77 | 28.41 | 60.18 | 59.29 | 5.37 | 5.41 |
|  | (30.72) | (20.00) | (27.34) | (29.95) | (40.42) | (57.82) | (44.52) |
|  |  |  |  |  |  |  |  |
| Observations | 96,436 | 96,873 | 96,875 | 96,344 | 97,181 | 103,654 | 103,656 |
| Adj. R-squared | 0.1387 | 0.1142 | 0.1911 | 0.0928 | 0.1199 | 0.0907 | 0.1075 |

Panel G: 4 year after

|  | (1) | (2) | (3) | (4) | (5) | (6) | (7) |
| --- | --- | --- | --- | --- | --- | --- | --- |
|  | *General health*  (SF-36) | *Vitality*  (SF-36) | *Mental health*  (SF-36) | *Role-emotional*  (SF-36) | *Social functioning* (SF-36) | *Satisfied with*  *Life?* | *Satisfied with health?* |
|  |  |  |  |  |  |  |  |
| *DEATH* | -0.81 | 2.78 | 1.31 | 1.43 | 0.87 | 0.04 | 0.01 |
|  | (-2.52) | (9.00) | (4.93) | (2.81) | (2.41) | (1.91) | (0.41) |
| *DEATH*×*FEMALE* | -1.99 | -6.61 | -4.33 | -6.44 | -5.54 | -0.09 | -0.25 |
|  | (-4.38) | (-15.32) | (-11.65) | (-8.77) | (-10.68) | (-3.04) | (-5.82) |
| *Age* | -0.37 | -0.13 | -0.09 | -0.30 | -0.28 | -0.01 | -0.04 |
|  | (-36.60) | (-13.63) | (-10.32) | (-17.48) | (-23.96) | (-19.89) | (-38.74) |
| *Married* | 2.87 | 1.17 | 3.63 | 7.32 | 5.44 | 0.35 | 0.23 |
|  | (10.85) | (4.65) | (16.90) | (17.16) | (17.94) | (19.54) | (9.54) |
| *Remote* | 0.37 | 0.93 | 1.08 | 0.53 | 1.47 | 0.13 | 0.09 |
|  | (1.06) | (2.81) | (3.71) | (0.94) | (3.74) | (5.48) | (2.88) |
| *Relative socio-economic disadvantage Index* (*decile*) | 0.42 | 0.26 | 0.41 | 0.38 | 0.55 | 0.04 | 0.04 |
|  | (4.52) | (2.92) | (5.56) | (2.53) | (5.19) | (6.38) | (4.94) |
| *Index of economic resources* (*decile*) | 0.04 | -0.14 | -0.12 | 0.17 | 0.16 | -0.03 | -0.01 |
|  | (0.45) | (-1.84) | (-1.88) | (1.40) | (1.81) | (-5.71) | (-1.55) |
| *Index of education and occupation* (*decile*) | 0.44 | 0.38 | 0.05 | 0.21 | 0.16 | 0.01 | 0.04 |
|  | (5.00) | (4.66) | (0.73) | (1.47) | (1.62) | (2.08) | (5.21) |
| *Personality scale - Agreeableness* | 0.62 | -0.12 | 0.59 | -1.04 | -0.17 | 0.16 | 0.07 |
|  | (3.78) | (-0.77) | (4.27) | (-3.93) | (-0.89) | (13.71) | (4.73) |
| *Personality scale - Conscientiousness* | 2.55 | 2.11 | 1.41 | 3.65 | 2.18 | 0.09 | 0.16 |
|  | (17.47) | (15.33) | (11.83) | (15.60) | (13.05) | (9.29) | (12.18) |
| *Personality scale - Emotional stability* | 4.05 | 4.06 | 5.56 | 5.09 | 4.16 | 0.20 | 0.28 |
|  | (28.27) | (29.74) | (47.39) | (22.22) | (25.21) | (20.30) | (20.31) |
| *Personality scale - Extroversion* | 1.64 | 2.57 | 2.36 | 1.36 | 1.54 | 0.13 | 0.11 |
|  | (13.06) | (21.40) | (22.34) | (6.74) | (10.35) | (15.35) | (9.51) |
| *Personality scale - Openness to experience* | -0.52 | -0.99 | -0.90 | -3.17 | -2.01 | -0.11 | -0.09 |
|  | (-3.64) | (-7.33) | (-7.79) | (-13.80) | (-12.39) | (-10.76) | (-7.03) |
| *Religion* | -0.32 | -0.24 | -1.09 | -2.08 | -2.14 | -0.01 | 0.02 |
|  | (-1.27) | (-1.02) | (-5.22) | (-5.07) | (-7.45) | (-0.44) | (1.06) |
| *Aborigines or Torres Strait Islander* | -5.32 | 2.38 | 1.06 | -15.41 | -4.75 | 0.37 | 0.37 |
|  | (-1.28) | (0.94) | (0.37) | (-1.91) | (-1.23) | (1.47) | (1.22) |
| *Education* | -0.45 | -0.05 | -0.14 | -0.30 | -0.44 | 0.01 | -0.03 |
|  | (-9.19) | (-1.15) | (-3.51) | (-3.89) | (-7.87) | (4.20) | (-6.60) |
| Constant | 39.22 | 24.33 | 29.47 | 60.47 | 61.59 | 5.44 | 5.68 |
|  | (30.11) | (20.09) | (28.13) | (29.26) | (42.16) | (60.49) | (46.77) |
|  |  |  |  |  |  |  |  |
| Observations | 92,785 | 93,216 | 93,217 | 92,705 | 93,517 | 100,040 | 100,041 |
| Adj. R-squared | 0.1360 | 0.1125 | 0.1841 | 0.0860 | 0.1104 | 0.0863 | 0.1006 |

This table presents the weighted OLS regression result on the difference between numerous measures capturing the respondents’ vitality level, mental health, and general health, role emotional and social functioning after matching groups of respondents who had experienced death of a friend in the past year against the respondents’ socio-demographics including age, marital status, ethnicity (ATSI), level of education, remoteness, personality traits, religion, socio-economic disadvantage, economic resources, and education and occupation. Non-bereaved group is reweighted using the Entropy Balancing (EB) procedure so that the distribution (mean, variance and skewness) of the socio-demographic variables are matched to the bereaved group. The dependent variables include the Short Form 36 Questionnaire (SF-36) scores on the respondent’s vitality, mental health, general health, role limitations due to emotional problems and social functioning (transformed into a scale from 0 to 100, where 0 is poor and 100 is excellent), how satisfied they are with their life and health (ranging from 0 to 10). In order to isolate the interdependency between gender and social activity, we also match the level of social activity in addition to the respondents’ socio-demographics. We report the coefficient of the dummy variable DEATH, which equals 1 if the respondent experienced death in the relevant time period. We also report coefficient of the interaction variable DEATH×FEMALE, where FEMALE is a dummy variable equals 1 if the respondent is a female. *t*-statistics are reported in parenthesis.
